# Comparison of doses and NTCP to risk organs with enhanced inspiration gating and free breathing for left-sided breast cancer radiotherapy using the AAA algorithm

**DOI:** 10.1186/s13014-015-0375-y

**Published:** 2015-04-10

**Authors:** Anneli Edvardsson, Martin P Nilsson, Sousana Amptoulach, Sofie Ceberg

**Affiliations:** Department of Medical Radiation Physics, Lund University, Lund, Sweden; Department of Oncology and Radiation Physics, Skåne University Hospital, Lund, Sweden; Department of Oncology and Radiation Physics, Skåne University Hospital, Malmö, Sweden

## Abstract

**Background:**

The purpose of this study was to investigate the potential dose reduction to the heart, left anterior descending (LAD) coronary artery and the ipsilateral lung for patients treated with tangential and locoregional radiotherapy for left-sided breast cancer with enhanced inspiration gating (EIG) compared to free breathing (FB) using the AAA algorithm. The radiobiological implication of such dose sparing was also investigated.

**Methods:**

Thirty-two patients, who received tangential or locoregional adjuvant radiotherapy with EIG for left-sided breast cancer, were retrospectively enrolled in this study. Each patient was CT-scanned during FB and EIG. Similar treatment plans, with comparable target coverage, were created in the two CT-sets using the AAA algorithm. Further, the probability of radiation induced cardiac mortality and pneumonitis were calculated using NTCP models.

**Results:**

For tangential treatment, the median V_25Gy_ for the heart and LAD was decreased for EIG from 2.2% to 0.2% and 40.2% to 0.1% (p < 0.001), respectively, whereas there was no significant difference in V_20Gy_ for the ipsilateral lung (p = 0.109). For locoregional treatment, the median V_25Gy_ for the heart and LAD was decreased for EIG from 3.3% to 0.2% and 51.4% to 5.1% (p < 0.001), respectively, and the median ipsilateral lung V_20Gy_ decreased from 27.0% for FB to 21.5% (p = 0.020) for EIG. The median excess cardiac mortality probability decreased from 0.49% for FB to 0.02% for EIG (p < 0.001) for tangential treatment and from 0.75% to 0.02% (p < 0.001) for locoregional treatment. There was no significant difference in risk of radiation pneumonitis for tangential treatment (p = 0.179) whereas it decreased for locoregional treatment from 6.82% for FB to 3.17% for EIG (p = 0.004).

**Conclusions:**

In this study the AAA algorithm was used for dose calculation to the heart, LAD and left lung when comparing the EIG and FB techniques for tangential and locoregional radiotherapy of breast cancer patients. The results support the dose and NTCP reductions reported in previous studies where dose calculations were performed using the pencil beam algorithm.

## Background

Although the use of adjuvant radiotherapy for breast cancer reduces the risk of local and locoregional recurrence as well as breast cancer death [1,2], some radiation is inevitably delivered to the heart and lungs, and for older radiotherapy techniques, an increased risk of cardiac mortality has been observed in radiotherapy for left-sided breast cancer [3]. However, studies evaluating more modern radiotherapy techniques, have been inconclusive regarding the increased cardiac mortality and morbidity for left-sided breast cancer radiotherapy [3-6]. A recent study indicated an increased risk of cardiac mortality and morbidity with increased mean absorbed dose to the heart, with no apparent threshold dose [7]. The left anterior descending (LAD) coronary artery is located in the anterior part of the heart, and is therefore likely to be exposed to high absorbed dose in breast radiotherapy [8]. Higher incidence of coronary artery disease has been seen among women irradiated for left-sided breast cancer, especially for LAD related disease [9]. According to Demirci et al. [10] the follow-up duration for more modern radiotherapy techniques is too short for any firm conclusions to be drawn and they therefore recommend that care should continue to be taken to minimize cardiac exposure. Until there is evidence of a threshold absorbed dose below which there is no excess risk of cardiac mortality and morbidity, it seems appropriate to aim at minimizing the absorbed dose to the heart and LAD.

It has also been shown that the risk of lung complications increases with increased absorbed lung dose [3,11]. For women who developed lung cancer after breast cancer radiotherapy, the lung cancer mortality for ipsilateral lung cancer was higher than from contralateral lung cancer [3] and the incidence of radiation pneumonitis has been shown to increase with increased absorbed lung dose [11].

In a recent review [12] different cardiac sparing techniques such as breathing adapted radiotherapy (BART), prone patient positioning, intensity modulated radiotherapy, proton beam radiotherapy and partial breast radiotherapy were evaluated. Several studies show that different forms of BART, such as enhanced inspiration gating (EIG) and deep inspiration breath hold (DIBH), can reduce the absorbed dose to the heart and lung, while keeping the same target coverage [13-18] and as a consequence of such dose reduction, the cardiac and pulmonary complication probabilities can be reduced [19]. During inspiration the spatial distance between the target volume and the heart is increased, excluding the heart and LAD from the high-dose regions. By only irradiating during the end-inspiration phase of the breathing cycle the absorbed dose to the heart and LAD can be decreased. At the same time the lung density is decreased, reducing the relative lung volume irradiated. Hence BART provides a possibility to reduce the cardiopulmonary dose without compromising target coverage.

In our clinic, EIG with audio-coaching has been in clinical use since 2007. All left-sided breast cancer patients, intended to be treated with EIG, have been subjected to both a conventional and a gated CT-scan to decide if they benefit from EIG and consequently should be treated with the technique.

According to Knöös et al. [20], treatment planning algorithms can be divided into ‘type a’ and ‘type b’. In ‘type b’ algorithms approximate modelling of lateral electron transport is included, which is not accounted for in ‘type a’ algorithms. ‘Type a’ algorithms include pencil beam (PB) algorithms and ‘type b’ include the Collapsed Cone (CC) and Anisotropic Analytical Algorithm (AAA). Fogliata et al. [21] showed that PB algorithms are defective in calculations involving lung, and even more defective in calculations involving low density lung, as in the case for deep inspiration. However, the calculation accuracy using ‘type b’ algorithms are much higher and the dose calculation accuracy is less affected by respiratory phase. Most previous treatment planning studies evaluating EIG and DIBH for left-sided breast cancer have used ‘type a’ algorithms, which do not properly account for lung heterogeneities. To our knowledge, only few studies have used ‘type b’ algorithms [22,23].

The purpose of this treatment planning study was to investigate the potential dose reduction to the heart, LAD and ipsilateral lung using EIG compared to free breathing (FB), using the AAA algorithm. The radiobiological implication of this dose difference, in the form of normal tissue complication probability (NTCP), was also investigated.

## Methods

### Ethical consideration and consent

The use of the radiotherapy database for retrospective research has been approved by the committee of the Regional Ethical Review Board in Lund (No. 2013/742). This research was waived informed consent.

### Patient selection

Thirty-two patients, who all received adjuvant radiotherapy for left-sided breast cancer using audio-coached EIG [22], were randomly selected and retrospectively enrolled in this study. The patients began their treatment between January and December 2011. Sixteen patients received tangential breast irradiation to the whole breast only after lumpectomy, nine of the patients received locoregional treatment after lumpectomy and seven patients received locoregional treatment after mastectomy, to represent all patients receiving radiotherapy for breast cancer. The median age of the patients was 46 (range 40–56) years.

### Respiratory gating

During EIG, the patients breathe deeper than normal, following individually adjustable inhale (3.6 to 5.2 s in this study) and exhale times (3.6 to 5.3 s in this study). Unlike for DIBH, where longer breath holds are used, the patients do not perform normal breathing between the deep breaths. The real-time positioning management system (RPM™, Varian Medical Systems, Palo Alto, CA) was used to monitor the patients’ breathing. This system consists of a marker block, with six reflective markers, placed on the chest of the patient, and infrared light reflected by these markers is detected by a camera to monitor the anteroposterior movement of the box. The marker block was positioned on the sternum, slightly to the right to avoid irradiating through the box. The image acquisition and irradiation was automatically turned on in a preselected interval of the breathing cycle, referred to as the gating window. Gating in the end-inspiration phase of enhanced free breathing based on the respiration amplitude was used. The patients were audio-coached during a training session (approximately 30 minutes), CT-scanning, set-up imaging and the radiotherapy treatment.

### CT-scanning

Both EIG and FB CT scans were acquired for all 32 patients. For 27 patients a 2-slice GE HiSpeed Nx/i Pro (GE Healthcare, Madison, WI) was used and for five patients a 64-slice Siemens Somatom definition AS plus (Siemens Medical Solutions, Erlangen, Germany) was used. The slice thickness was 3 mm and the acquisition was made in axial scan mode for EIG and helical scan mode for FB. The patients were positioned in a standard breast board (Posiboard-2, Civco Medical Solution, IA, USA) with both arms above the head. The CT scanning was automatically started when the breathing curve entered the gating window.

### Delineation of structures

Structures were delineated in both the EIG and FB CT-sets by two radiation oncologists. The two oncologists delineated all structures however they divided the work so that a specific structure in all CT sets was delineated by the same oncologist. For tangential breast irradiation to the whole breast only after lumpectomy, the PTV was defined as the clinical limits of the breast, with a minimum of 10 mm margin to all glandular tissue. The CTV-T was defined as the volume where the tumor had been located, approximately equivalent to a quadrant of the breast. For locoregional treatment after lumpectomy, the PTV was defined as the clinical limits of the breast, ipsilateral axillary lymph nodes level I-III, and supra- and infraclavicular fossa. The CTV-T was defined as above. For locoregional treatment after mastectomy, the PTV was defined as the part of the thoracic wall were the breast had been located (visualized on CT scans by markers), ipsilateral axillary lymph nodes level I-III, and supra- and infraclavicular fossa. No CTV-T was delineated for these patients. The PTV was cropped 5 mm from the skin surface. The organs at risk (OAR) delineated were the heart, LAD and ipsilateral lung. The heart and LAD were manually delineated whereas the ipsilateral lung was automatically delineated using the segmentation wizard in the treatment planning system (TPS, Eclipse, version 10.0, Varian Medical Systems, Palo Alto, CA) and then manually verified. The heart was defined as the entire myocardium, excluding the pericardium where a distinction could be made, starting superiorly at the beginning of pulmonary trunc and aorta. LAD was delineated starting from the exit of left coronary artery (which was thus included) from aorta, continuing in the anterior interventricular sulcus down to as close to the apex as the sulcus could be visualized. All OARs were delineated without margins.

### Treatment planning

The treatment planning was carried out by one physicist, on the basis of the guidelines provided by the Swedish breast cancer group [24]. According to these national guidelines, dose coverage of the PTV should be prioritized higher than the OARs for lobular and multifocal breast cancer. Otherwise the constraints and guideline values for the heart and lung dose should be prioritized higher than dose coverage of the PTV. Dose coverage of the CTV-T should always be first priority. In this study, dose coverage of the PTV was always prioritized over the absorbed dose to the OARs regardless of the patients’ diagnosis. The main goals of the treatment plans were that 100% of the CTV-T volume should be covered by 95% of the prescribed dose (V_95%,CTV-T_ =100%), 100% of the PTV volume should be covered by 93% of the prescribed dose (V_93%,PTV_ =100%) and the volume receiving more than 105% of the prescribed dose (V_105%_) should be minimized. At the same time the absorbed dose to the OARs was kept as low as possible. Although the national guidelines were not completely followed, this way of performing the treatment planning gave the opportunity to evaluate the possible decrease in doses to risk organs, if the dose coverage of the PTV was prioritized higher than the OAR constraints. Regarding the arrangement of the treatment beams, essentially identical plans were created in both the EIG and FB CT-images. Only minor differences in the placement of the additional fields, gantry angle and field weight were allowed to get comparable target coverage between the two plans. The absorbed dose was normalized to the PTV mean dose and the calculation algorithm used was the AAA version 10.0.28. The prescribed dose was 50 Gy in 25 fractions. Three-dimensional conformal treatment planning using a single isocenter technique was used.

For tangential treatment planning, two tangential 6 MV photon fields with a posterior PTV margin of 5 mm were used. For dose homogenization, 10 or 18 MV fields, with the same shape as the 6 MV fields, were added for some of the patients. For the same reason, additional smaller fields, with lower field weight, were also added for all of the patients. All fields were conformed using the Millenium multileaf collimator (Varian Medical Systems, Palo Alto, CA) with a central and peripheral leaf width of 5 and 10 mm, respectively.

For locoregional treatment planning, the PTV was divided into a cranial and caudal part with the isocenter placed in the junction. The treatment planning of the caudal part was carried out in the same way as for tangential treatment, with at least one additional field, with lower field weight, covering the junction. For the cranial part, anterior and posterior photon fields were used. For the anterior field the energy used depended on the location of the target. Also a mixture of different energies was used. For the posterior field, the highest energy was always used (10 or 18 MV). A posterior field with lower field weight, shielding for the lung, was also added.

### NTCP calculation

The probability for cardiac mortality and radiation pneumonitis was calculated using the relative seriality model [25]:

1 $$ NTCP={\left\{1-{{\displaystyle {\prod}_{i=1}^n\left[1-P{\left({D}_i\right)}^s\right]}}^{\varDelta {V}_i}\right\}}^{\raisebox{1ex}{$1$}\!\left/ \!\raisebox{-1ex}{$s$}\right.} $$

2 $$ P\left({D}_i\right)={2}^{- exp\left\{e\gamma \left(1-{D}_i/{D}_{50}\right)\right\}} $$

where D_i_ is the absorbed dose in each dose bin, i, of the differential dose volume histogram (DVH), D_50_ is the dose resulting in 50% complication probability, γ is the maximum relative slope of the dose–response curve, n is the number of DVH dose bins, ΔV_i_ = V_i_/V where V_i_ is the volume of the each dose bin and V is the total volume of the organ. The relative seriality factor, s (range 0 to 1), describes the tissue architecture. Input data for the NTCP calculations with endpoint excess cardiac mortality was taken from Gagliardi et. al. [26] for the entire heart volume: s = 1, γ = 1.28 and D_50_ = 52.3 Gy. For the endpoint radiation pneumonitis input data was taken from Gagliardi et al. [11], corrected for the use of the AAA algorithm by the use of algorithm-specific NTCP parameters determined by Hedin et al. [27]: s = 0.012, γ = 0.974 and D_50_ = 27.52 Gy for tangential treatment and s = 0.012, γ = 0.966 and D_50_ = 29.23 Gy for locoregional treatment.

### Data analysis

**Figure 1:**
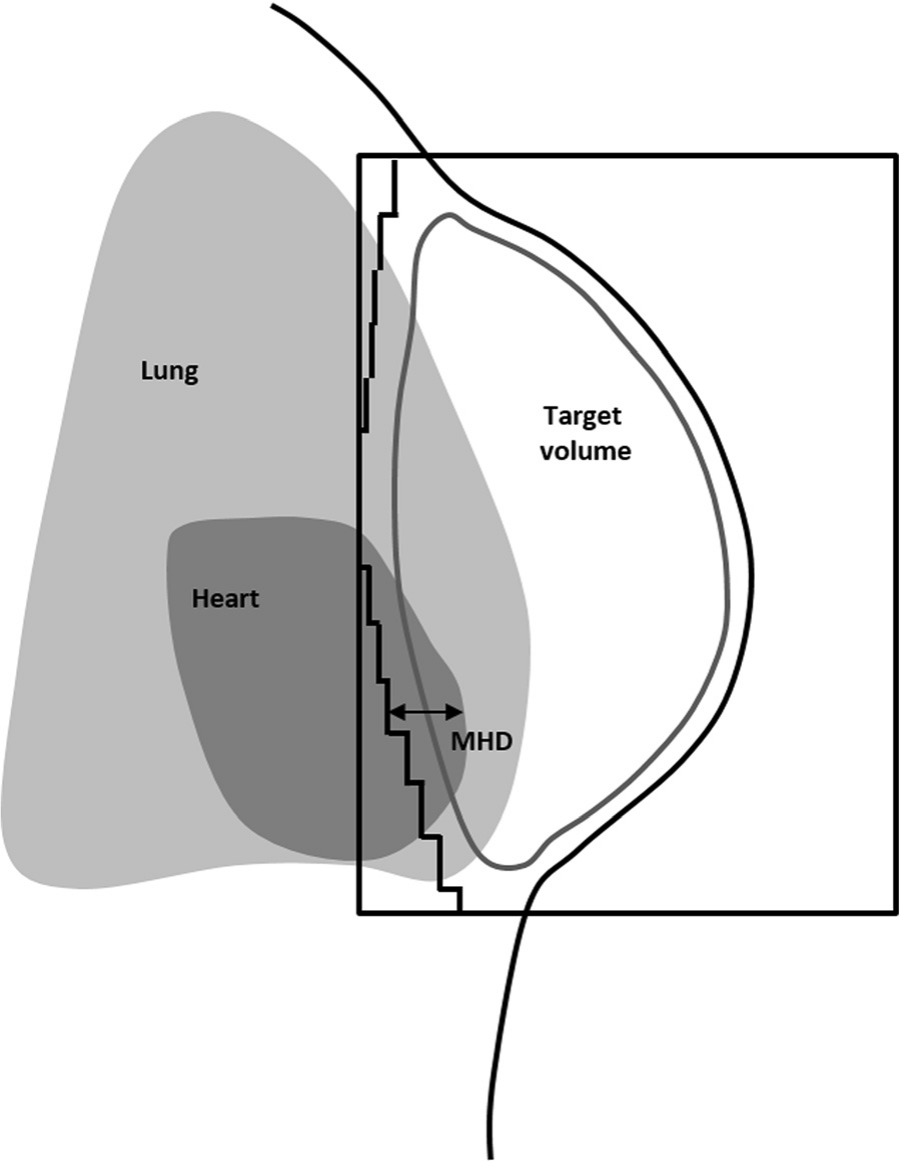
**Definition of the maximum heart distance.** The maximum heart distance (MHD) is defined as the maximal distance between the contour of the heart and the posterior MLC of a tangential field.

DVHs, with a dose bin size of 0.05 Gy, were retrieved from the TPS. For the heart and LAD, the mean absorbed dose (D_mean,heart/LAD_), the near maximum absorbed dose (the dose to 2% of the volume, D_2%,heart/LAD_) and the volume receiving more than 25 Gy (V_25Gy,heart/LAD_) were compared between EIG and FB. For the ipsilateral lung the mean absorbed dose (D_mean,lung_) and the volume receiving more than 20 Gy (V_20Gy,lung_) were compared. Also the CTV-T volume covered by 95% of the prescribed dose (V_95%,CTV-T_) and the PTV volume covered by 93% of the prescribed dose (V_93%,PTV_) were compared. Additionally, the structure volumes (V_PTV/CTV-T/heart/LAD/lung_) were retrieved from the TPS and the maximum heart distance (MHD) was measured in the beam’s eye view. The MHD is the maximal distance between the contour of the heart and the posterior MLC of a tangential field (Figure 1). The breathing amplitudes during the CT session were retrieved from the RPM system.

Two-sided paired Wilcoxon tests were carried out to evaluate the difference between the two treatment techniques. Two-sided unpaired Wilcoxon tests were carried out to evaluate the difference between the tangential and locoregional groups of patients. Values of p < 0.01 were considered statistically significant.

## Results

**Figure 2:**
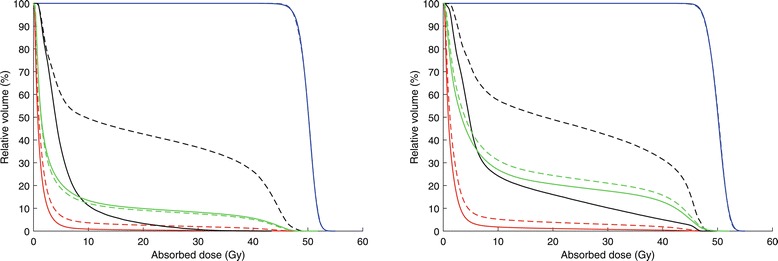
**Mean dose volume histograms.** Mean dose volume histograms for tangential treatment (left) and locoregional treatment (right) comparing EIG (solid lines) and FB (dashed lines) for LAD (black), heart (red), ipsilateral lung (green) and PTV (blue).

For both tangential and locoregional treatment, the D_mean,heart_, D_mean,LAD_, D_2%,heart_, D_2%,LAD_, V_25Gy,heart_ and V_25Gy,LAD_ were significantly decreased for EIG compared to FB (p < 0.001) (Table 1, Figure 2). Also the MHD was significantly decreased for both tangential and locoregional treatment (p < 0.001). Based on NTCP calculations, the excess cardiac mortality probability was significantly decreased (p < 0.001) for EIG compared to FB for both tangential and locoregional treatment (Table 2).

**Table 1:** **Treatment planning data for target and organs at risk for free breathing (FB) and enhanced inspiation gating (EIG) for tangential and locoregional treatment, presented as median values, range in brackets and p-values for paired Wilcoxon tests**

	**Tangential treatment**			**Locoregional treatment**		
	**FB**	**EIG**	**p**	**FB**	**EIG**	**p**
V_PTV_ (cm^3^)	891 [290-2622]	888 [297-2557]	0.469	1136 [325-2670]	1092 [308-2656]	0.278
V_93%,PTV_ (%)	98.7 [96.9-99.2]	98.2 [96.8-99.4]	0.017	98.2 [96.4-99.3]	98.3 [96.4-99.3]	0.255
V_CTV-T_ (cm^3^)	40 [16-215]	43 [17-217]	<0.001	40 [6-93]	41 [6-93]	0.492
V_95%,CTV-T_ (%)	100.0 [98.0-100.0]	100.0 [98.9-100.0]	0.019	99.9 [95.8-100.0]	99.9 [95.7-100.0]	0.469
V_heart_ (cm^3^)	593 [387-771]	580 [399-747]	0.438	613 [447-783]	615 [455-743]	0.255
D_mean,heart_ (Gy)	2.5 [1.3-4.4]	1.3 [0.8-2.1]	<0.001	3.1 [1.9-5.4]	1.5 [1.0-3.8]	<0.001
D_2%,heart_ (Gy)	28.5 [6.1-44.2]	5.2 [3.2-21.0]	<0.001	38.4 [9.6-46.1]	5.5 [3.8-41.7]	<0.001
V_25Gy,heart_ (%)	2.2 [0.3-6.3]	0.2 [0.0-1.7]	<0.001	3.3 [0.9-7.6]	0.2 [0.0-4.1]	<0.001
MHD* (cm)	1.3 [0.5-2.4]	0.4 [0.0-1.8]	<0.001	1.7 [0.9-2.9]	0.6 [0.0-1.9]	<0.001
V_LAD_ (cm^3^)	1.8 [1.1-2.8]	1.8 [1.0-3.0]	0.313	1.7 [1.0-3.3]	1.8 [0.7-3.1]	0.234
D_mean,LAD_ (Gy)	18.5 [3.5-39.8]	5.5 [2.4-9.3]	<0.001	25.4 [8.7-33.1]	8.0 [4.1-30.6]	<0.001
D_2%,LAD_ (Gy)	44.7 [8.6-48.8]	16.7 [3.7-34.6]	<0.001	46.1 [27.8-48.9]	28.9 [6.5-46.9]	0.002
V_25Gy,LAD_ (%)	40.2 [0.0-87.7]	0.1 [0.0-7.2]	<0.001	51.4 [3.5-75.8]	5.1 [0.0-62.4]	<0.001
V_lung_ (cm^3^)	1132 [786-1617]	1765 [1224-2325]	<0.001	1047 [626-1249]	1683 [1080-2410]	<0.001
D_mean,lung_ (Gy)	5.4 [2.1-9.4]	5.5 [2.6-9.1]	0.215	14.0 [6.4-18.9]	11.2 [6.1-16.4]	0.002
V_20Gy,lung_ (%)	9.1 [2.2-17.2]	9.1 [2.9-17.2]	0.109	27.0 [8.7-38.8]	21.5 [7.9-31.9]	0.020

*Maximum heart distance.

**Table 2:** **Excess cardiac mortality probability and risk of radiation pneumonitis in percent for tangential and locoregional treatment, presented as median values, range in brackets and p-values for paired Wilcoxon tests**

	**Tangential treatment**			**Locoregional treatment**		
	**FB**	**EIG**	**p**	**FB**	**EIG**	**p**
Excess cardiac mortality probability	0.49 [0.03-1.74]	0.02 [0.00-0.37]	<0.001	0.75 [0.12-2.14]	0.02 [0.00-1.01]	<0.001
Risk of radiation pneumonitis	0.31 [0.04-1.99]	0.38 [0.05-1.78]	0.179	6.82 [0.47-17.72]	3.17 [0.41-11.51]	0.004

For tangential treatment, there was no significant difference in D_mean,lung_ and V_20Gy,lung_ between EIG and FB (p = 0.215 and p = 0.109, respectively) (Table 1, Figure 2). Further, there was no significant difference (p = 0.179) in the risk of clinical pneumonitis between EIG and FB (Table 2). For locoregional treatment, however, there was a statistically significant decrease in D_mean,lung_ for the ipsilateral lung for EIG compared to FB (p = 0.002) (Table 1, Figure 2) whereas there was no significant difference in V_20Gy,lung_ (p = 0.020). The risk of clinical pneumonitis was significantly decreased (p = 0.004) for EIG compared to FB for locoregional treatment (Table 2).

The median breathing amplitude for EIG during the CT session was 7.0 (5.1-14.4) mm for tangential treatment and 6.9 (4.9-11.4) mm for locoregional treatment (p = 0.522), and hence the breathing amplitudes during EIG were comparable for the tangential and locoregional groups of patients. Comparisons of the structure volumes and target coverage are presented in Table 1.

## Discussion

Several previous studies have emphasized the limitations of ‘type a’ dose calculation algorithms in treatment situations including lung tissue. In general, due to the fact that lateral electron transport is not scaled appropriately, ‘type a’ algorithms will overestimate the coverage of the target volumes and underestimate the low dose volumes in nearby risk organs. Since the effect is smaller in the 50% dose region, the dose to risk organs has previously often been reported in terms of V_50%_ [14]. The range effect is expected to be larger if the lung density is lower, and it has been concluded that these types of algorithms are not suitable for comparing treatment techniques where the lung density varies, such as during BART for breast cancer [13,14,18,19]. The AAA algorithm, which is used in this study, is known to give more accurate calculation results in low density volumes, such as the lungs, compared to the PB algorithm [21]. Especially, the accuracy is increased in the low-dose and the high-dose regions. This is important for NTCP calculations, which requires information of the entire DVH. In this study, we have replaced the previously used NTCP parameters, which were derived using a PB algorithm according to the studies by Gagliardi et al. [11,26], by algorithm-specific parameters for the endpoint radiation pneumonitis, as determined by Hedin et al. [27]. Good calculation accuracy in the high-dose region is also important for estimating the effects on the LAD, for which an increased incidence of coronary artery stenosis have been associated with high doses [9].

Radiotherapy for breast cancer is an adjuvant therapy, used to reduce the recurrence rate and increase survival. According to Darby et al. and Clarke et al. [1,2], one recurrence is avoided for approximately every fifth patient irradiated for breast cancer and one breast cancer death is avoided for approximately every twentieth patient irradiated. Consequently, due to the lack of predictive methods the majority of the breast cancer patients will not benefit from the radiotherapy treatment. Additionally, this is also a large group of patients with an expected long-time survival, which emphasizes the importance to keep the long-time side effects as low as possible. The results from this study show that EIG significantly reduce the heart and LAD absorbed doses and the excess cardiac mortality probability for both tangential and locoregional treatment and the ipsilateral lung absorbed dose and risk of clinical pneumonitis for locoregional treatment. These results support previously published studies using PB algorithms showing that EIG reduces doses to risk organs and NTCP [14,19].

Comparing tangential and locoregional treatment, the potential to reduce the absorbed dose to the heart and LAD is higher using EIG for locoregional treatment. The absolute reduction in the median excess cardiac mortality probability was 0.73 percentage points for locoregional treatment and 0.47 percentage points for tangential treatment, implying a larger absolute sparing in excess cardiac mortality probability using EIG for locoregional treatment than for tangential treatment. Also, the absorbed dose to the ipsilateral lung and excess risk of radiation pneumonitis were significantly decreased for locoregional treatment, but not seen for tangential treatment. The internal mammary nodes (IMN) were not included in the target in this study, following clinical practice. Inclusion of the IMNs in the target implies higher doses to the heart and ipsilateral lung, and hence potentially larger dose reduction to OARs using BART. In the studies by Korreman et al. [14,19] and Hjelstuen et al. [18] the IMNs were included in the target and the absorbed doses to the heart, LAD and ipsilateral lung were higher compared to this study.

This study confirms, using the AAA algorithm, that EIG can reduce the absorbed dose to the heart and LAD and the cardiac mortality probability shown in previous studies using PB algorithms [14,19]. Furthermore, this study shows that this is also the case although IMNs are excluded in the target. Also the absorbed dose to the ipsilateral lung was decreased for locoregional treatment but not for tangential treatment. Available studies report conflicting results regarding the absorbed dose for the ipsilateral lung for tangential treatment using BART [13,15-17]. A possible reason for this could be that larger breathing amplitudes (18 mm in [13] and 10.9 mm for EIG and 21.3 mm for DIBH in [17]) were used in these studies which resulted in a decreased lung dose compared to the present study. Damkjær et al. [22] showed that a smaller lung volume was irradiated to high absorbed dose using DIBH compared to EIG due to a larger breathing amplitude for this treatment technique. Hence, increased breathing amplitudes could possibly result in decreased ipsilateral lung dose and NTCP also for tangential treatment. Larger breathing amplitudes are also required to completely remove the heart from the treatment fields, especially for locoregional treatment due to the higher MHD for this group (Table 1). For 6 out of the 16 patients receiving tangential treatment and 4 out of the 16 patients receiving locoregional treatment, the heart was completely outside the treatment fields for EIG. However, the volume receiving high absorbed dose (V_25Gy_) was reduced for all except one of the patients. For none of the patients the heart was completely outside the treatment fields for FB. Since the heart can be considered to be a serial organ for the endpoint cardiac mortality [26], possibly due to irradiation of the coronary arteries, a reduction of the maximum dose to the heart and coronary arteries is of great importance.

There were no significant differences in V_93%,PTV_ or V_95%,CTV-T_ for neither tangential nor locoregional treatment. The volume of CTV-T was significantly larger for EIG compared to FB for tangential treatment. The reason for this is not known. The difference was rather small and is not believed to affect the result of the comparison. Except for the expected increase of the ipsilateral lung volume, there were no other significant differences in the structure volumes. Hence the structure volumes and treatment plans can be considered comparable between FB and EIG. Comparable target coverage is crucial to be able to compare the absorbed dose to the OARs.

The challenges in defining the heart volume and the subregions of the heart have been pointed out by the QUANTEC group [28]. Lorenzen et al. [29] showed a large uncertainty in the estimated absorbed dose to especially LAD but also to the heart due to inter-observer variations in the delineation of these structures. No contrast was used for visualization of LAD in this study and therefore the whole LAD was not distinguishable in the CT images, leading to uncertainties in the delineation of LAD. However, LAD was similarly delineated by the same oncologist in the EIG and FB CT sets and the uncertainty in LAD delineation is only believed to have minor impact on the comparison of these two techniques. However, this might affect the comparison of the results in this study with other studies.

The parameters used to calculate the cardiac mortality probability was determined assuming a homogeneous radiation sensitivity within the heart. The dose reduction observed with EIG occurs primarily in the anterior part of the heart where LAD is positioned and therefore a greater reduction of long-term ischemic disease might be expected. The parameters used to calculate the cardiac mortality probability in this study are based on older radiotherapy techniques and may not reflect the radiotherapy techniques of today. Also, the NTCP parameters are based on data with higher incidence of excess cardiac mortality than calculated in the present study. Hence the magnitude of the heart NTCP should not be interpreted as exact, however for the purpose of this study, to compare two different treatment techniques it gives a reasonable estimate of the complication probability for the two techniques.

According to the QUANTEC group [30] approximately 1–5% of the patients irradiated for breast cancer develop clinically significant symptomatic radiation pneumonitis. The NTCP calculations of the risk of radiation pneumonitis presented in this study (Table 2), using the parameters by Gagliardi et al. [11] corrected for the use of the AAA algorithm by the use of algorithm-specific NTCP parameters determined by Hedin et al. [27], are in close agreement with the risk of developing radiation pneumonitis according to the QUANTEC group.

According to the guidelines by the Swedish breast cancer group [24], sparing of the heart should be prioritized over the target coverage for all but lobular and multifocal breast cancer, which means that target coverage is compromised against sparing of the OARs. However, due to the increased distance between the breast and the heart during EIG, the target coverage does not have to be compromised. Consequently, the gain from EIG could be reduced OAR absorbed doses or increased target coverage with the same OAR doses or a combination of the two. In this study, the target was always prioritized over the OARs and hence this study demonstrates the OAR dose-sparing possibility using EIG.

Until the risk of cardiac mortality and morbidity from modern radiotherapy techniques for breast cancer is better known it seems reasonable that the absorbed dose to the heart should be kept as low as possible. The QUANTEC group [28] recommends that for patients with breast cancer the irradiated heart volume should be minimized as much as possible without compromising target coverage. This is shown to be possible for EIG using the AAA algorithm in this study, confirming the dose sparing of risk organs shown in previously published research using PB algorithms.

## Conclusions

Using the AAA algorithm for dose calculation, enhanced inspiration gating significantly decreases the absorbed dose to the heart and left anterior descending coronary artery without compromising the target coverage, for both tangential and locoregional treatment, resulting in decreased cardiac mortality probability. The absorbed dose to the ipsilateral lung was significantly decreased for locoregional treatment, resulting in decreased radiation pneumonitis probability for this patient group. The results support the dose and NTCP reductions reported in previous studies where dose calculations were performed using the pencil beam algorithm.
